# A Partial Picture of the Single-Cell Transcriptomics of Human IgA Nephropathy

**DOI:** 10.3389/fimmu.2021.645988

**Published:** 2021-04-16

**Authors:** Rong Tang, Ting Meng, Wei Lin, Chanjuan Shen, Joshua D. Ooi, Peter J. Eggenhuizen, Peng Jin, Xiang Ding, Jinbiao Chen, Yangshuo Tang, Zhou Xiao, Xiang Ao, Weisheng Peng, Qiaoling Zhou, Ping Xiao, Yong Zhong, Xiangcheng Xiao

**Affiliations:** ^1^ Department of Nephrology, Xiangya Hospital, Central South University, Changsha, China; ^2^ Department of Pathology, Xiangya Hospital, Central South University, Changsha, China; ^3^ Department of Hematology, the Affiliated Zhuzhou Hospital Xiangya Medical College, Central South University, Zhuzhou, China; ^4^ Centre for Inflammatory Diseases, Monash University, Clayton, VIC, Australia; ^5^ Department of Organ Transplantation, Xiangya Hospital, Central South University, Changsha, China; ^6^ Department of Medical Records & Information, Xiangya Hospital, Central South University, Changsha, China; ^7^ Department of Ultrasound, Xiangya Hospital, Central South University, Changsha, China

**Keywords:** IgA nephropathy, single-cell RNA sequencing, kidney, TNF and IL-17 signaling, NOD-like receptor signaling

## Abstract

The molecular mechanisms underlying renal damage of IgA nephropathy (IgAN) remain incompletely defined. Here, single-cell RNA sequencing (scRNA-seq) was applied to kidney biopsies from IgAN and control subjects to define the transcriptomic landscape at single-cell resolution. We presented a comprehensive scRNA-seq analysis of human renal biopsies from IgAN. We showed for the first time that IgAN mesangial cells displayed increased expression of several novel genes including MALAT1, GADD45B, SOX4, and EDIL3, which were related to cell proliferation and matrix accumulation. The overexpressed genes in tubule cells of IgAN were mainly enriched in inflammatory pathways including TNF signaling, IL-17 signaling, and NOD-like receptor signaling. Furthermore, we compared the results of 4 IgAN patients with the published scRNA-Seq data of healthy kidney tissues of three human donors in order to further validate the findings in our study. The results also verified that the overexpressed genes in tubule cells from IgAN patients were mainly enriched in inflammatory pathways including TNF signaling, IL-17 signaling, and NOD-like receptor signaling. The receptor-ligand crosstalk analysis revealed potential interactions between mesangial cells and other cells in IgAN. IgAN patients with overt proteinuria displayed elevated genes participating in several signaling pathways compared with microproteinuria group. It needs to be mentioned that based on number of mesangial cells and other kidney cells analyzed in this study, the results of our study are preliminary and needs to be confirmed on larger number of cells from larger number of patients and controls in future studies. Therefore, these results offer new insight into pathogenesis and identify new therapeutic targets for IgAN.

## Introduction

IgA nephropathy (IgAN) is the most common primary glomerular disease worldwide (1, 2). IgAN takes a slow but relentless clinical course which eventually progresses to end-stage renal disease (ESRD) in 30–40% of patients within 20–30 years of diagnosis (3).

There is now strong evidence that IgAN is an autoimmune disease, and a multi-hit hypothesis has been proposed to explain the immunopathogenesis of IgAN (4–6). Other mechanisms including mesangial- podocytic-tubular crosstalk, genetic factors, and complement activation also participate in pathogenesis of IgAN (6, 7). However, comprehensive analysis of the cell types and molecular pathways that promote disease progression is lacking.

Traditional bulk RNA sequencing (RNA-seq) is a powerful tool to profile transcriptomic variations in different diseases. However, bulk RNA-seq only evaluates the average gene expression of a large population of cells in the tissue (8). The fast development of single-cell RNA sequencing (scRNA-seq) technology allows the inquiry of transcriptomic profiles and signaling pathways in diverse cell types from a given sample simultaneously, and unlike bulk RNA-seq, it can define comprehensive gene sets at the single-cell level (9, 10). Recently, this new methodology has been employed in various renal diseases (11–14). In the present study, we applied scRNA-seq to kidney samples from patients with IgAN to identify gene expression at the single cell level, and explore novel cellular interactions and crucial molecular pathways contributing to the disease development.

## Methods

### Ethical Approval and Consent

Samples were collected as part of the Kidney Precision Medicine Study which was approved by the Medical Ethics Committee of the Xiangya Hospital of Central South University for Human Studies (approval number 201711836). All subjects provided written informed consent, and all experiments were performed in accordance with study protocol.

### Public Dataset Acquisition and Processing

The scRNA-Seq data of healthy kidney tissues of three human donors were downloaded from the Gene Expression Omnibus (GSE131685). We reproduced the downstream analysis using the code provided by the author in the original paper. We applied Harmony to integrate samples and performed downstream analysis using Seurat (version 3.1, Satija Lab, https://satijalab.org/seurat/). All gene expression was normalized and scaled using NormalizeData and ScaleData. Top 2,000 variable genes were selected by FindVariableFeautres for PCA analysis. Clustering analysis using FindClusters was performed by first reducing the gene expression matrix to the first 20 principal components and then using a resolution of 0.3 for graph-based clustering.

### Clinical Sample Procurement

Kidney specimens from four newly diagnosed IgAN patients were obtained from department of nephrology in Xiangya Hospital, Central South University. Kidney sample as a portion of renal core needle biopsies by 18-gauge needles indispensable for clinical diagnosis was obtained from consented IgAN patients. Control kidney tissues were collected at one site by needle biopsy of living donor kidneys after removal from the donor and before implantation to the recipient. Only small amount (2–3 mg) of renal biopsy was acquired for the scRNA-seq procedure. Kidney tissues were cleaned with sterile PBS after obtainment.

### Kidney Sample Processing and Single-Cell Dissociation

The fresh kidney sample was placed into the GEXSCOPE Tissue Preservation Solution (Singleron Biotechnologies) immediately at 2–8°C (15). After washed by Hanks Balanced Salt Solution (HBSS) for three times, biopsy sample was cut into small pieces (1–2 mm). Subsequently, sample was digested in 2 ml GEXSCOPE Tissue Dissociation Solution (Singleron Biotechnologies) in a 15 ml centrifuge tube by continuous agitation maintained at 37°C for 15 min. Sample was then filtered through a 40-μm sterile cell strainer (Corning). After centrifuged at 1,000 rpm for 5 min at 4°C, cell pellets were resuspended with 1 ml phosphate buffer saline (PBS) (HyClone). The cell suspension was incubated with 2 ml GEXSCOPE Red Blood Cell Lysis Buffer (Singleron Biotechnologies) for 10 min at 25°C to remove red blood cell. After centrifuged at 1,000 rpm for 5 min, the cell pellet was resuspended with PBS. Then cells were counted using a TC20 automated cell counter (Bio-Rad). Live cells were determined by trypan blue staining (Gibco). If the cell viability exceeded 85%, the subsequent sample processing was performed.

### Library Preparation and Preprocessing of scRNA-Seq Data

The single-cell suspension was adjusted to a concentration of 1 × 10^5^ cells/ml in PBS. Subsequently, single cell suspension was loaded onto the microfluidic chip. The single cell RNA-seq libraries were prepared following the manufacturer’s protocols (Singleron GEXSCOPE Single Cell RNA-seq Library Kit, Singleron Biotechnologies) (15). The captured single cell RNA-seq libraries were sequenced were sequenced by using an Illumina HiSeq X10 instrument and 150 bp paired-end reads.

### Marker Genes Analysis in Different Cell Types

After cell cluster identification, the marker genes of each cell population in kidney were determined relative to other cell clusters with the “Wilcox” (Likelihood-ratio test) using the FindAllMarkers function in Seurat. The selected marker genes were expressed in over 10% of the cells in one cluster and average log (Fold Change) was more than 0.25. The heatmap was completed by the top 20 marker genes of each cell cluster.

### Differentially Expressed Genes Identification Between Groups

We identified differentially expressed genes (DEGs) in each cell cluster of kidney by comparing the transcriptional profile of IgAN and control subjects. The DEGs of each cell cluster between the two groups were defined with the "wilcox" (Likelihood-ratio test) by the FindAllMarkers function in Seurat. Avg.exp replied the average expression of the gene in the cell type. The gene with absolute value of average log (Fold Change) exceeded 0.25 and *P* value smaller than 0.05 was identified as differentially expressed gene.

### Enrichment and Cell Interaction Analysis

Gene ontology (GO) and Kyoto encyclopedia of genes and genomes (KEGG) enrichment analysis were performed by “cluster Profiler” R package. We also conducted cell the analysis of cell interactions *via* Cellphone DB.

## Results

A total of five renal biopsy specimens were collected from patients with IgAN (n = 4), and healthy control (n = 1) obtained from living donor kidney. All four IgAN patients had proteinuria ranged from 0.27 to 2.57 g per 24 h. IgAN subjects were divided into microproteinuria and overt proteinuria group. Microproteinuria which was determined as daily urinary protein excretion amount 20–300 mg (16), was observed in one IgAN patient (25%). Overt proteinuria which was defined as daily urinary protein excretion exceeding 300 mg (16), was observed in three IgAN subjects (75%). The estimated glomerular filtration rate (eGFR) of IgAN patients ranged from 32.72 to 114.09 ml/min/1.73m^2^ (81.83 ± 38.07 ml/min/1.73m^2^). Serum IgA concentration was elevated in one patient (**Supplemental Table 1**). IgAN subjects had no coexistent diseases such as diabetes and lupus. Other secondary kidney diseases were also excluded. At the time of biopsy, all IgAN patients have not received any medications including RAAS blockers in the past. The information about blood pressure was shown in **Supplemental Table 1**. The living relative donor was a 41-year-old male, with no history of diseases including hypertension and diabetes. His creatinine was 0.94 mg/dl, and eGFR was 100.47 ml/min/1.73m^2^. He has not been prescribed any medications in the past.

### Cell Lineage in Kidney Identified by scRNA-seq

**Figure 1:**
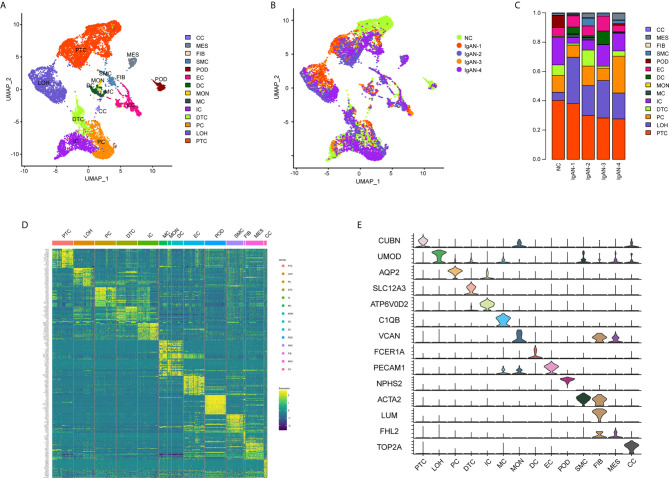
Cell lineage analysis by comprehensive single-cell RNA-sequencing in IgAN and control subjects. **(A)** Fourteen distinct cell clusters identified by UMAP plotting, and clusters were colored and labeled distinctively. The color of cells represented the group origin. **(B)** UMAP plot of cell clusters from different subjects of IgAN patients (n = 4) and control (n = 1). The color of cells reflected the individual origin. **(C)** Bar plots represented frequency of cell clusters in kidneys from different subjects. Blocks represented different subjects, and block height was in proportion to the number of cells. **(D)** Heatmap of top 20 specific marker genes for each cluster from kidney. Each column represented a kind of cell cluster, and each row corresponded to marker gene for individual cluster. **(E)** Violin plot of selected marker genes that identified the clusters generated by UMAP plotting. It was colored by different cell subtypes. Abbreviations were as follows: PTC, proximal tubule cells; LOH, loop of Henle cells; PC, principal cells; IC, intercalated cells; DTC, distal tubule cells; EC, endothelial cells; POD, podocytes; MES, mesangial cell; SMC, smooth muscle cells; DC, dendritic cells; MC, macrophages; MON, monocytes; CC, cycling cells; FIB, fibroblasts.

A total of 20,570 cells were isolated and sequenced in five specimens. The cell viability ranged from 85 and 91% (**Supplemental Table 2**). We performed data pre-processing and quality control, then got the transcriptomic data. Fourteen distinct cell clusters were defined using unsupervised clustering analysis, and labeled according to lineage-specific marker gene expression (**Figure 1A**). Enrichment of different cell clusters was calculated for each subject respectively (**Figure 1B**). The numbers of cells in each cluster in kidney from individual patients and control were provided in **Supplemental Table 8**. Bar plots represented frequency of cell clusters in kidney of each subject (**Figure 1C**). The expression of top 20 marker genes for each cell population among 14 clusters proved the reliability of cell classification method (**Figure 1D** and **Supplemental Table 7**). **Figure 1E** and **Table 1** showed each cell population identified by selected cell lineage-specific marker gene expression.

**Table 1:** Cell-lineage-specific marker genes of different cell types.

Cell type	Marker genes
Mesangial cells (MES)	FN1, FHL2, CTGF, MYL9
Endothelial cells (EC)	PECAM1, VWF, CLDN5, ACKR1
Phagocytes (POD)	NPHS2, PODXL, PTPRO
Proximal tubular cells (PT)	CUBN, SLC13A1, LRP2, ALDOB
Distal tubular cells (DT)	SLC12A3, CALB1, SLC8A1
Loop of Henle cells (LOH)	UMOD, SLC12A1, CLDN16
Principal cells (PC)	AQP2, AQP3
Intercalated cells (IC)	SLC26A7, SLC4A1, ATP6V1G3
Macrophages (MC)	LYZ, CD68, MRC1, C1QA, C1QB
Monocytes (MON)	LYZ, VCAN, CD14, FCN1
Dendritic cells (DC)	CD1C, CD1E, FCER1A, CLEC10A
Fibroblasts (FIB)	LUM, COL1A1, DCN
Smooth muscle cells (SMC)	ACTA2, TAGLN, MYH11, MYLK
Cycling cells (CC)	TOP2A, MKI67

### Identification of Gene Expression Changes in Glomerulus of IgAN Subjects

**Figure 2:**
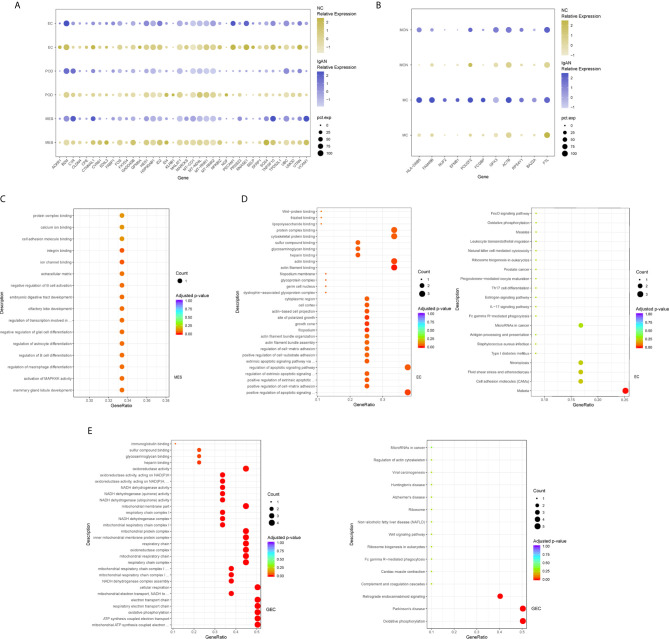
DEGs and intercellular signaling in glomerulus and immune cells of IgAN and control subjects. **(A)** Representative DEGs in mesangial cells, endothelial cells and podocytes in kidney by comparing the transcriptional profile of IgAN and control subjects. **(B)** Representative DEGs in monocytes and macrophages by comparing the transcriptional profile of IgAN and control subjects. **(C)** GO enrichment analysis displayed that upregulated DEGs were involved in some biological processes in mesangial cells comparison with control subject. **(D, E)** GO and KEGG enrichment analysis revealed DEGs were mainly enriched in some biological processes and pathways in endothelial cells and glomerular endothelial cells respectively. Abbreviations were as follows: pct.exp, percentage of cells expressing gene; count, number of genes annotated to GO terms or KEGG pathway.

We next compared transcriptomes of intrinsic renal cells from glomerulus in IgAN patients with healthy counterpart. Entire lists of cell-type-specific DEGs were displayed in **Supplemental Table 3**. We defined representative DEGs in mesangial cells, endothelial cells, and podocytes through comparing the transcriptional profile of IgAN and control subjects (**Figure 2A**). Detailed information about DEGs in each cell cluster in kidney from IgAN and control subjects were displayed in **Supplemental Table 9**.

Overexpression of several genes (*MALAT1*, *GADD45B*, *SOX4*, and *EDIL3*) in IgAN mesangial cells were reported here for the first time. *MALAT1*, a highly conserved nuclear long non-coding RNA molecule, which is implicated in extracellular matrix (ECM) production, oxidative stress, and fibrosis (17), was upregulated in IgAN mesangial cells. *GADD45B*, a member of the growth arrest and DNA damage related gene family, has been proved to play significant roles in DNA damage, cell growth, and apoptosis (18). *GADD45B* probably participated in podocytes injure of focal segmental glomerular sclerosis (FSGS) (19). The effects of tumor related genes (*SOX4* and *EDIL3*) in IgAN need further clarification. *FOS* is a member of the *FOS* gene family which acts as a regulator of cell proliferation, differentiation, and apoptosis (20). One recent study demonstrated that *FOS* expression was enriched in IgAN patients with important roles in mesangial proliferation and glomerular sclerosis (21). Consistently, gene expression profiles showed *FOS* expression was elevated in mesangial cells of IgAN. GO enrichment analysis showed DEGs in the mesangial cells were enriched for biologic processes including integrin binding and ion channel binding (**Figure 2C**).

In order to further validate the findings in our study, we compared the results of four IgAN patients with the published kidney cell atlas data downloaded from the Gene Expression Omnibus (GSE131685). As shown in **Supplemental Table 10**, compared with our previous data, there were more highly expressed DEGs in IgAN mesangial cells and endothelial cells. Consistent with our previous findings, we also detected overexpression of several genes (GADD45B, FOS, ID2, and MT-RNR1) in IgAN mesangial cells. Furthermore, several genes (SOX4, MT-RNR1, PECAM1, UTRN, MTATP6P1, and MT-ND4L) were also found to be upregulated in IgAN endothelial cells.

As shown in **Figure 2A**, IgAN podocytes had increased expression of the serine protease *PRSS23*, which has been demonstrated to be highly expressed in the glomeruli of human fibrotic kidneys and may play a pathogenic role in renal fibrosis (22). *NGF*, a protective factor overexpressed in the kidney of various renal disorders (23), was elevated in podocytes of IgAN. As a growth factor, *NGF* protects immune and non-immune cells from apoptotic cell death and might be involved in kidney physiopathology in previous findings (23). IgAN podocytes expressed increased *HES1*, which acts as an effector of Notch signaling pathway. It was reported previously that *HES1* expression in podocytes mediated epithelial to mesenchymal transition (EMT) (24). We found that DEGs included overexpressed cellular adhesion gene (*SELP* and *PECAM1*) and angiogenesis (*XIPS*) in glomerular endothelial cells of IgAN. IgAN endothelial cells also expressed increased levels of the transcription factor *SOX4*, a central component of TGF-β signaling to mediate EMT in various types of cancer (25), which has not been reported in IgAN yet. The elevated expression of atypical chemokine receptor *ACKR1* was displayed in IgAN endothelial cells. *ACKR1*, which is enriched within endothelial junction, has a role in supporting leukocyte recruitment (26). Interestingly, endothelial cell cluster highly expressed the endothelial extracellular endonuclease *RNASE1*, acting as a key regulator to maintain endothelial homeostasis and protect the endothelial layer (27). Furthermore, endothelial cells were divided into glomerular endothelial cells and vascular endothelial cells. GO and KEGG enrichment analysis showed DEGs in the endothelial cells were enriched for the biologic processes or signaling including positive regulation of cell-matrix adhesion, apoptotic signaling, and oxidation (**Figures 2D, E**).

### Tubular Cells From IgAN Patients Were Enriched With Inflammatory, Matrix Remodeling and Related Signatures

**Figure 3:**
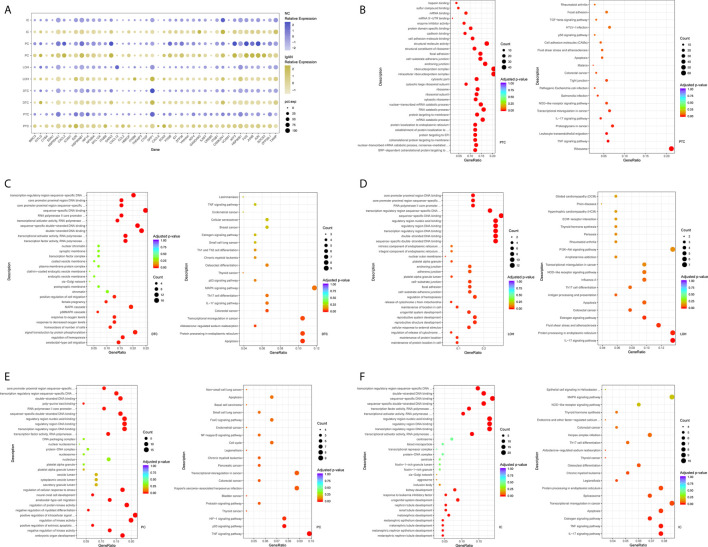
DEGs and intercellular signaling in tubules of IgAN and control subjects. **(A)** Representative DEGs in proximal tubule cells, distal tubule cells, loop of Henle cells, principal cells, and intercalated cells by comparing the transcriptional profile of IgAN and control subjects. **(B)** GO enrichment analysis displayed that upregulated DEGs in proximal tubule cells were enriched in some biological processes, KEGG analysis showed these genes in proximal tubule cells were mainly involved in the pathways, such as TNF signaling, IL-17 signaling, and NOD-like receptor signaling pathway. **(C–F)** GO and KEGG enrichment analysis of DEGs in distal tubule cells, loop of Henle cells, principal cells, and intercalated cells respectively comparing IgAN to control subjects. Abbreviations were as follows: pct.exp, percentage of cells expressing gene; count, number of genes annotated to GO terms or KEGG pathway.

The epithelial cells were obtained and sub-clustered into five groups based on marker genes. We also identified representative DEGs in renal tubules in IgAN and control subjects (**Figure 3A** and **Supplemental Table 4**). Altered signaling networks in the tubular cells were identified in IgAN subjects. Comparison of proximal tubule cells from IgAN and control subjects revealed upregulation of genes participating in TNF signaling, IL-17 signaling, NOD-like receptor signaling, and regulating leukocyte transendothelial migration (**Figure 3B**). Comparison of the DEGs in the distal tubule cells revealed enrichment of genes participating in MAPK cascade, p38MAPK cascade in IgAN (**Figure 3C**). The loop of Henle cells in IgAN had increased genes participating in TNF signaling, IL-17 signaling, Th17 cell differentiation, NOD-like receptor signaling. Moreover, genes participating in PI3K-Akt signaling, including *ITGB6*, *ITGB8*, and *YWHAH*, were increased in the loop of Henle cells in IgAN. Also, genes participating in Toll-like receptor signaling, including *SPP1*, *JUN*, and *FOS* were increased in the loop of Henle cells in IgAN (**Figure 3D**).

As illustrated in **Figures 3E, F**, principal cells and intercalated cells were enriched for genes participating in TNF signaling, IL-17 signaling, Th17 cell differentiation in IgAN. Furthermore, *NFKBIA*, *TXNIP*, *CXCL3*, and *CXCL2*, which were all get involved in NOD-like receptor signaling regulation (28), were increased both in principal cells and intercalated cells. Further studies are needed to elucidate NOD-like receptor signaling in pathogenesis of IgAN.

As shown in **Supplemental Tables 11** and **12**, we compared the results of four IgAN patients with the published kidney cell atlas data in order to further validate the findings in our study. The tubular epithelial cells were sub-clustered into five groups. Many altered signaling networks in renal tubular cells were also identified and validated in IgAN subjects. Interestingly, comparison of the DEGs in the proximal tubular cells from IgAN patients also revealed enrichment of genes participating in TNF signaling, IL-17 signaling, and NOD-like receptor signaling. Distal tubular cells from IgAN subjects had elevated expression of genes participating in IL-17 signaling. The loop of Henle cells in IgAN also had increased genes participating in TNF signaling, IL-17 signaling, Th17 cell differentiation, NOD-like receptor signaling, and PI3K-Akt signaling. Consistently, principal cells were also enriched for genes participating in TNF signaling, IL-17 signaling, and NOD-like receptor signaling in IgAN.

### Identification of Gene Expression Changes in Immune Cells in Kidney of IgAN Subjects

We identified leukocytes which were composed of macrophages, monocytes, and dendritic cells in kidney sample of IgAN patients, which was consistent with previous research (29). However, significant numbers of some leukocyte subpopulations including T cells in kidney were not detected, that might be a potential limitation due to either dissociation technical limitation and/or the cell number below our detection limit, as the infiltration and effect of renal T cells in pathogenesis of IgAN is well-proven (30). Because few numbers of leukocytes appear in control, we used two peripheral blood mononuclear cell (PBMC) datasets specimens commonly available as control (31, 32).

Dataset integration showed DEGs of monocyte and macrophage subsets (**Supplemental Table 5**). *GPX3*, a member of glutathione peroxidase family, which could protect cells against oxidative stress (33), was reduced in renal macrophages of IgAN. As illustrated in **Figure 2B**, macrophages from kidney in IgAN displayed decreased expression of *FAM49B*, which is primarily localized in the mitochondria and its downregulation leads to increased fission and mitochondrial reactive oxygen species (ROS) production (34). *FCGBP*, a negative regulator of EMT, which is proved to have the function of cell protection and anti-inflammation in tissues (35), was under-expressed in macrophages from kidney of IgAN.

### Comparative Analysis Identify Cell-Cell Crosstalk in IgAN Through Ligand-Receptor Interactions Analysis

**Figure 4:**
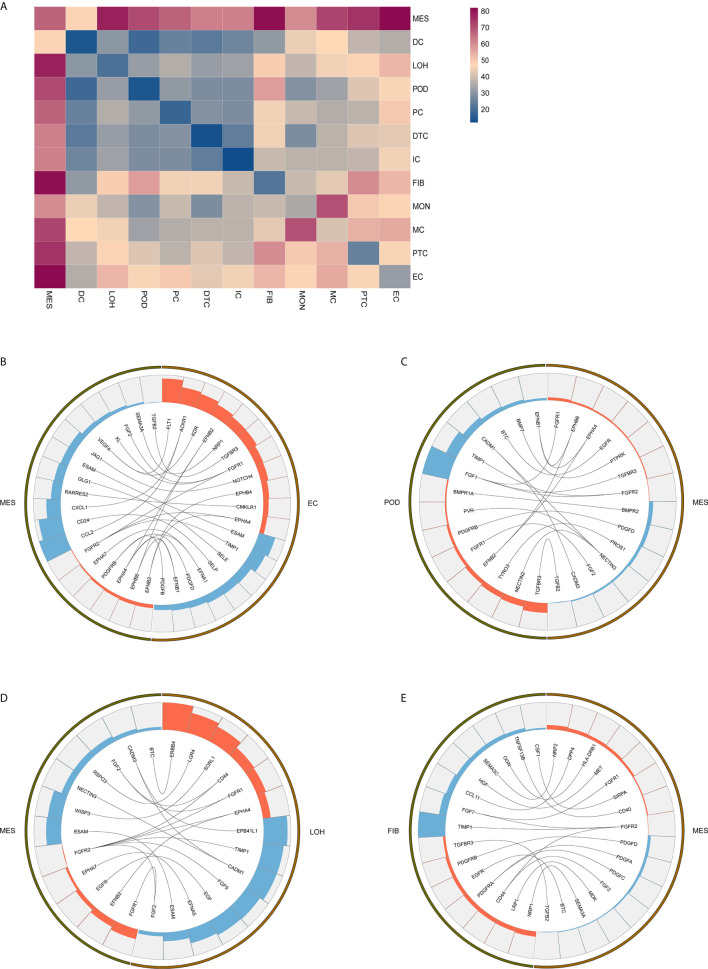
Possible ligand-receptor interactions between different cell types in the kidney of IgAN patients. **(A)** Ligand-receptor signaling pathways between cell clusters in the kidney were analyzed and shown. **(B)** Representative ligand-receptor interactions between mesangial cells and endothelial cells. **(C)** Representative ligand-receptor interactions between mesangial cells and podocytes. **(D)** Representative ligand-receptor interactions between mesangial cells and loop of Henle cells. **(E)** Representative ligand-receptor interactions between mesangial cells and fibroblasts. Lines represented interrelations between the mesangial cells and other cells. Lines between the ligand and conjuncted receptor were shown. Each cell type was depicted by colors. Only IgAN patients (n = 4) were analyzed.

Exploring the intercellular communication and signaling network may provide new opportunities to identifying new therapeutic targets in IgAN. Potential interactions of receptors and ligands in distinct cell types of kidney were presented (**Figure 4A**). IgAN mesangial cells expressed *CXCL1* and a potent monocyte-attracting chemokine *CCL2*. *CXCL1*, a member of the CXC chemokine family, serves as a mesangial-derived adjuster to induce damage of other cells in IgAN, other than its neutrophil chemoattractant ability (36). We found *CXCL1* and *CCL2* expressed in mesangial cells might both interact with the chemokine receptor *ACKR1* expressed at endothelial junctions. In addition, mesangial cells expressed *JAGGED1*, and its receptor *NOTCH4*, was expressed in endothelial cells, indicating involvement of Jagged/Notch signaling pathways in pathogenesis of IgAN (**Figure 4B**). Mesangial cells expressed the growth factors *FGF2* and *PDGFD*, which promote proliferation and matrix-production of mesangial cells by interacting with their respective receptors *FGFR1* and *PDGFRB* expressed in podocytes or Loop of Henle (**Figures 4C, D**). *PDGFRB*, indicating a potential mechanism for mesangial proliferation and renal fibrosis (37), was also expressed in fibroblasts (**Figure 4E**). Similarly, ligand-receptor pair analysis could be performed for the remaining interactions between different cells of interest for further study.

### DEGs and Pathway Analysis in Kidney From IgAN Patients With Overt Proteinuria Compared With IgAN Patients With Microproteinuria

The gene expression profile was compared between IgAN subjects with microproteinuria and overt proteinuria. Detailed information of cell-type-specific DEGs in two groups of IgAN was shown in **Supplemental Table 6**. The gene expression profile in kidney of microproteinuria group was different from that of overt proteinuria group. *SPARC*, an extracellular matrix-associated glycoprotein, was significantly increased in mesangial cells of overt proteinuria group, compared to microproteinuria group. Overexpression of *SPARC* has been shown to colocalize with collagen, and participate in extracellular matrix deposition and renal fibrosis (38). IgAN patients with overt proteinuria exhibited higher mesangial expression of *ROCK2*, compared with microproteinuria group, indicating it might be a new potential therapeutic target for IgAN. Previously, *ROCK2* has been proved to promote mesangial proliferation and ECM production by strengthening the inflammatory process and fibrotic circuitry in diabetic nephropathy (39). Furthermore, in comparison to microproteinuria group, overt proteinuria group showed upregulated endothelial *TXNIP* expression, which facilitates oxidative stress and inflammatory response (40). Also, *SPARCL1* and *CD74*, which are involved in the regulation of cell adhesion, migration, and proliferation, were higher in endothelial cells of the overt proteinuria group compared to the microproteinuria group (41, 42).

**Figure 5:**
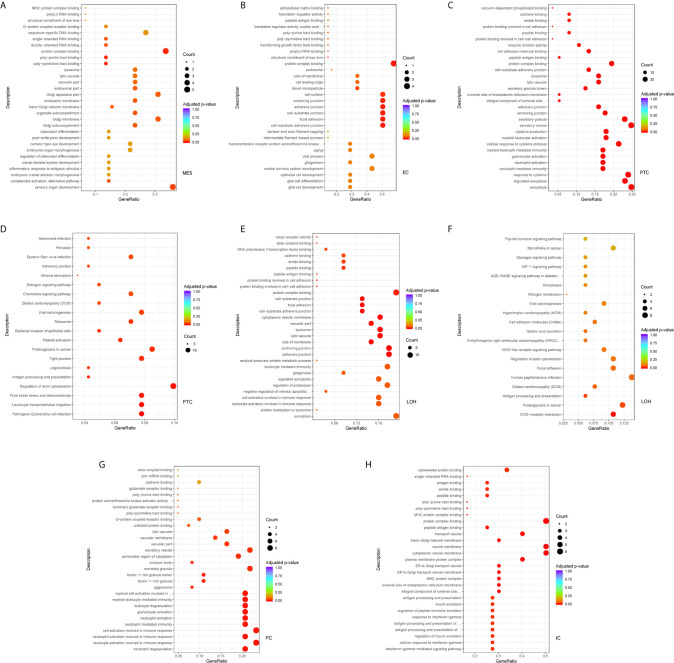
DEGs involved in biological process and intercellular signaling in kidney from IgAN patients with overt proteinuria compared to microproteinuria. **(A, B)** GO enrichment analysis in mesangial cells and endothelial cells respectively between IgAN patients with overt proteinuria and with microproteinuria. **(C–F)** GO and KEGG enrichment analysis in proximal tubule cells and loop of Henle cells. **(G, H)** GO enrichment analysis in principal cells and intercalated cells between IgAN patients with overt proteinuria and with microproteinuria. Abbreviations were as follows: count, number of genes annotated to GO terms or KEGG pathway.

Comparison of the DEGs in mesangial cells displayed enrichment of genes participating in complement activation and alternative pathway in IgAN with overt proteinuria in comparison to microproteinuria (**Figure 5A**). Endothelial cells in IgAN with overt proteinuria had increased genes involved in extracellular matrix binding and cell-substrate adherens junction (**Figure 5B**). Furthermore, genes involved in many pathways including leukocyte transendothelial migration, chemokine signaling, and type I interferon signaling pathway were increased in proximal tubule cells of IgAN subjects with overt proteinuria (**Figures 5C, D**). The loop of Henle cells had elevated genes participating in ECM-receptor interaction, interferon-gamma-mediated signaling, and neutrophil activation (**Figures 5E, F**). Correspondingly, principal cells had elevated genes involved in granulocyte activation and p38MAPK cascade (**Figure 5G**). Intercalated cells had increased genes involved in interferon-gamma-mediated signaling pathway and T cell costimulation in IgAN with overt proteinuria compared with microproteinuria group (**Figure 5H**).

## Discussion

In this study, we comprehensively generated profiles of distinct cell types and gene expression in kidney biopsy specimen using scRNA-seq in IgAN subjects and living donor control. We also verified that scRNA-seq analysis of kidney sample obtained from IgAN patients was a feasible and effective technique. We presented a comprehensive scRNA-seq analysis of human renal biopsy tissue from IgAN. Our findings revealed upregulation of cell proliferation and cell adhesion related genes and activation of inflammatory pathways by comparing DEGs in different cell populations. Our study provided several novel insights into pathogenesis of human IgAN through detecting and analyzing cell subpopulations, cell-type-specific gene expression, and distinct signaling pathways.

Although the exact pathogenesis has not been fully elucidated, increased polymeric IgA deposition in the mesangium within the kidney is the characteristic of IgAN (2). Also, it is considered universally that hypercellularity of mesangial cells and mesangial expansions represent primary pathological mechanisms in the development of IgAN (43). In this study, we show for the first time, several upregulated genes (*MALAT1*, *GADD45B*, *SOX4*, and *EDIL3*) in IgAN mesangial cells. Their overexpression might be related to mesangial cells proliferation and matrix accumulation. Only few studies have reported that upregulation of *GADD45B* expression participating in podocytes injury of FSGS (19). There were no published studies about *MALAT1* in kidney diseases so far. These findings suggested mesangial expression of these genes might emerge as novel potential biomarker and therapeutic targets underlying IgAN.

Glomerular endothelial proliferation and adhesion are involved in progression of IgAN, and the upregulation of adhesion molecules in endothelial cells correlates with severity of glomerular inflammation in IgAN (44). The overexpressed genes in our investigation were mainly enriched in the processes of cell-matrix adhesion, leukocyte migration, and EMT. ScRNA-seq detected several genes which have not been reported in IgAN previously. Glomerular endothelial cells had upregulated genes of regulators of angiogenesis (*XIPS*), leukocyte adhesion molecules (*PECAM1*), EMT (*SOX4*), and leucocyte recruitment (*ACKR1*). Among them, *XIPS* was not reported previously in kidney pathologies. Together, these observations indicate that these glomerular endothelial cells alterations may be involved in pathogenesis of IgAN, the significance and specificity of these markers need further investigation to validate.

We also detected DEGs and related signaling pathways in tubule cells from IgAN subjects and control. A growing body of literature indicates that the tubulointerstitial inflammation and fibrosis are common features of chronicity and disease development, and renal prognosis correlates more closely with the degree of tubulointerstitial injury than the severity of glomerular lesions. The crucial role of tubular epithelial cells in IgAN has also been suggested (45). Our findings showed the overexpressed genes in proximal tubule cells were mainly enriched in TNF signaling, IL-17 signaling, and leukocyte transendothelial migration. TNF signaling pathway plays a crucial role in a variety of physiological and pathological processes, including cell proliferation, apoptosis, differentiation, induction of inflammation, and regulation of immune reactions, and it also acts as a pathogenic signaling in progression of IgAN (46, 47). T-cell-derived cytokine IL-17 and related signaling can activate downstream pathways including NF-kappa B and MAPKs to increase the expression of pro-inflammatory cytokines and chemokines (48). IL-17 signaling has been implicated in promoting inflammatory cytokines release, leukocytes recruitment, and progression of kidney injury in IgAN (49). Our group previously found that IgAN mice had increased elevated frequency of Th17 cells, and Th17-related cytokines including IL-17A and CCL20 were all increased in the kidneys (50). In our study, TNF signaling and IL-17 signaling were also observed in other tubule cells including loop of Henle cells, principal cells, and intercalated cells, indicating the effects of these signaling may be extensive in IgAN. Interestingly, we identified elevated genes participating in NOD-like receptor signaling in tubule cells including proximal tubule cells, loop of Henle cells, principal cells, and intercalated cells. NOD-like receptor signaling can translate danger recognition into the production of proinflammatory cytokines and chemokines, and play a critical role in human diseases (28). One previous study has shown that NLRP3, a member of NLRs, localized mainly to the tubular epithelium, and increased NLRP3 expression correlates with better clinical prognosis in IgAN patients (51). Furthermore, a prior study from our group showed that kidney tissue NLRC5 expression was significantly increased in the IgAN compared to that in the healthy control (52). Therefore, our novel finding about the upregulated genes involved in NOD-like receptor signaling will provide new insights into in pathogenesis of IgAN. Furthermore, in order to further validate the findings in our study, we compared the results of four IgAN patients with the published scRNA-Seq data of healthy kidney tissues of three human donors. The results also verified that the overexpressed genes in tubule cells from IgAN patients were mainly enriched in inflammatory pathways including TNF signaling, IL-17 signaling, and NOD-like receptor signaling.

By analyzing the receptor-ligand crosstalk among distinct cell types in kidney of IgAN, we determined intercellular signaling networks. For example, fibrotic related signaling and chemokine signaling pathway were observed in kidney of IgAN. We found mesangial cells expressed the growth factors *FGF2* and *PDGFD* indicative of a fibrotic reaction, which interacted with their respective receptors *FGFR1* and *PDGFRB* detected in resident kidney cells including podocytes and fibroblasts. The FGF and PDFG pathways are associated with ECM accumulation and renal fibrotic processes (37). We also found chemokines *CXCL1 and CCL2* in mesangial cells interacted with their chemokine receptor *ACKR1* expressed in endothelial junctions, implying renal resident cells cross-talk may serve as modulator for immune cells recruitment and infiltration in the kidney. Further confirmation of these interactions would be important for developing novel therapeutic targets.

In order to explore molecular signatures in IgAN patients with different degrees of proteinuria, we examined transcriptomics of different cell clusters in kidney. Genes involved in mesangial proliferation and ECM production (*SPARC*, *ROCK2*) were upregulated in mesangial cells from IgAN subjects with overt proteinuria compared to microproteinuria group. DEGs analysis of kidney also revealed that higher expression of genes (*TXNIP*, *SPARCL1*, and *CD74*), which participated in cell adhesion, migration, and inflammation, were also displayed in endothelial cells of kidney from overt proteinuria group than microproteinuria group. Interestingly, we also found genes participating in diverse biological processes and signaling pathways including complement activation, leukocyte transendothelial migration, chemokine signaling, and type I interferon signaling pathway, were increased in diverse cell clusters of kidney from IgAN with overt proteinuria. Our dataset may indicate new mechanisms correlating with different degrees of proteinuria in the progression of IgAN.

Infiltration of immune cells in renal tissue of IgAN patients have been found in clinicopathologic studies previously (30). It has been reported that interstitial or glomerular immune cells, especially macrophages/monocytes accumulation are related to proteinuria and renal damage in IgAN (29). Similarly, our research revealed an elevated number of macrophages, monocytes, and dendritic cells in kidney samples of IgAN subjects. Compared with publicly available PBMC control, infiltrating IgAN macrophages have decreased expression of genes (*GPX3*, *FAM49B*, and *FCGBP*), which are cell protective factors mainly related with anti-oxidation and anti-fibrosis. Therefore, these alterations may elicit the oxidative and inflammatory response in kidney of IgAN.

However, our study had several limitations. First, the sample size in this research was relatively small. Increasing number of samples is needed to reflect the disease severity and stages, and limit the heterogeneity and individual variation in IgAN in future study. Second, we detected relatively few leukocytes and some resident glomerular cells such as podocytes, which might not reflect whole gene expression status at different parts and times. It might reflect dissociation bias of scRNA-seq technology. Third, the above novel findings were only indicated at the transcriptomic level in IgAN. Fourth, as no other kidney disease control group was included in this study, the changes of DEGs are not specific for IgAN and the changes may be generic to being proteinuria or glomerular inflammation. Fifth, based on number of mesangial cells and other kidney cells analyzed (**Supplemental Table 8**), the results of our study are preliminary and needs to be confirmed on larger number of cells from larger number of patients and controls in future studies. Furthermore, the results discovered in our study need further validation using tissue staining, functional studies *in vitro* using cell lines or primary human cells, and animal models of IgAN.

Collectively, this study showed cell-specific transcriptional profiles in kidney, and identified several novel genes, involved signaling pathway and potential pathologic ligand-receptor interaction in IgAN patients. ScRNA-seq of kidney tissues revealed new insights into molecular signatures and provided potential targets for the treatment of IgAN.

## Data Availability Statement

The original contributions presented in the study are publicly available. This data can be found here: Gene Expression Omnibus, GSE171314.

## Ethics Statement

The studies involving human participants were reviewed and approved by Medical Ethics Committee of the Xiangya Hospital of Central South University. The patients/participants provided their written informed consent to participate in this study.

## Author Contributions

YZ, RT, TM, WL, and XX conceived and designed the research; YZ, RT, JO, and XX wrote the paper; JO, PE, XA, and WP, QZ, and PX revised the paper; TM, WL, YZ, YT, and ZX carried out experiments; RT, TM, WL, and CS generated and provided analytical tools; JC, RT, YZ, XX, CS, PJ, and XD analyzed data. All authors contributed to the article and approved the submitted version.

## Funding

This work was funded by the National Key R&D Program of China (2020YFC2005000 to XX), the National Natural Science Foundation of China (81800641 to TM and 81500559 to RT), the Key Research and Development Program of Hunan province (2018WK2060 to XX and 2020WK2008 to YZ), the science and technology innovation Program of Hunan Province (2020RC5002 to JO), the Natural Science Foundation of Hunan Province (2018JJ3818 to RT), Chinese Society of Nephrology (18020010780 to YZ).

## Acknowledgments

Parts of the present study have been accepted as a Mini-Oral at the 58th ERA-EDTA Congress, which will be organised from June 5 to 8, 2021.

## Conflict of Interest

The authors declare that the research was conducted in the absence of any commercial or financial relationships that could be construed as a potential conflict of interest.

## Supplementary Material

Supplemental Table 1 Demographic and biochemical characteristics of patients with IgAN.

Supplemental Table 2 Number and viability of cells in kidney from each sample.

Supplemental Table 3 DEGs in cell subtypes of glomerulus comparing IgAN and control subjects. Abbreviations were as follows: pct.1, the percentage of cells where the gene is detected in the first group (IgAN patients); pct.2, the percentage of cells where the gene is detected in the second group (control subject); avg logFC, log fold-change of the average expression between the two groups, positive values indicate that the gene is more highly expressed in the first group.

Supplemental Table 4 DEGs in tubular cells from IgAN and control subjects.

Supplemental Table 5 DEGs in immune cells from IgAN and control subjects.

Supplemental Table 6 DEGs in different cell clusters of kidney from IgAN patients with overt proteinuria compared to microproteinuria.

Supplemental Table 7 Top 20 marker genes of each cell type shown in heatmap

Supplemental Table 8 Cell number of distinct clusters in kidney from each subject

Supplemental Table 9 Detailed information about DEGs in each cell cluster in kidney from IgAN and control subjects. Abbreviations were as follows: avg.exp, the average expression of the gene in the cell type; ave exp scaled, avg.exp after normalization; pct.exp, percentage of cells expressing gene; feature plot represented gene name.

Supplemental Table 10 DEGs by cell type comparing four IgAN patients with the published scRNA-Seq data of healthy kidney tissues of three human donors downloaded from the Gene Expression Omnibus (GSE131685).

Supplemental Table 11 Gene ontology enrichment analysis for individual cell types performed with the “cluster Profiler” R package using DEGs (**Supplementary Table 10**).

Supplementary Table 12 Kyoto encyclopedia of genes and genomes (KEGG) enrichment analysis for individual cell types performed with the “cluster Profiler” R package using DEGs (**Supplementary Table 10**).
